# Clinical results of proton beam radiotherapy for inoperable stage III non-small cell lung cancer: a Japanese national registry study

**DOI:** 10.1093/jrr/rrad017

**Published:** 2023-05-03

**Authors:** Hitoshi Tatebe, Hideyuki Harada, Keita Mori, Hiromitsu Iwata, Tetsuo Akimoto, Masao Murakami, Takahiro Waki, Takashi Ogino, Masatoshi Nakamura, Hiroshi Taguchi, Haruhiko Nakayama, Miyako Satouchi, Hidefumi Aoyama

**Affiliations:** Proton Therapy Center, Fukui Prefectural Hospital, 2-8-1 Yotsui, Fukui 910-0846, Japan; Radiation and Proton Therapy Center, Shizuoka Cancer Center, 1007 Shimonagamubo, Nagaizumi-cho, Sunto-Gun, Shizuoka 411-8777, Japan; Department of Biostatistics, Clinical Research Center, Shizuoka Cancer Center, 1007 Shimonagamubo, Nagaizumi-cho, Sunto-Gun, Shizuoka 411-8777, Japan; Department of Radiation Oncology, Nagoya Proton Therapy Center, Nagoya City University West Medical Center, 1-1-1 Hirate-cho, Kita-ku, Nagoya 462-8508, Japan; Division of Radiation Oncology and Particle Therapy, National Cancer Center Hospital East, Kashiwa 277-0882, Japan; Southern Tohoku Proton Therapy Center, 7-172, Yatsuyamada, Fukushima, Koriyama 963-8052, Japan; Department of Radiology, Tsuyama Chuo Hospital, 1756 Kawasaki, Tsuyama City, Okayama 708-0841, Japan; Medipolis Proton Therapy and Research Center, 4423 Higashikata, Ibusuki City, Kagoshima 891-0304, Japan; Department of Radiation Oncology, Faculty of Medicine, University of Tsukuba, 1-1-1 Tennodai, Ibaraki, Tsukuba 305-8577, Japan; Department of Radiation Oncology, Hokkaido University Hospital, Kita 15, Nishi 7, Kita-ku, Sapporo, Hokkaido 060-0808, Japan; Department of Thoracic Surgery, Kanagawa Cancer Center, 2-3-2 Nakao, Asahi-ku, Yokohama City, Kanagawa 241-8515, Japan; Department of Thoracic Oncology, Hyogo Cancer Center, 13-70 kitaojicho, Akashi, Hyogo 673-8558, Japan; Department of Radiation Oncology, Hokkaido University Faculty of Medicine, Hokkaido 060-0808, Japan

**Keywords:** particle beam therapy, proton beam therapy, chemoradiotherapy, inoperable stage III non-small cell lung cancer

## Abstract

This study presents the first data of a Japanese nationwide multi-institutional cohort and compares them with the findings of systematic literature reviews on radiation therapies and inoperable stage III non-small cell lung cancer (NSCLC) conducted by the Lung Cancer Working Group in the Particle Beam Therapy (PBT) Committee and Subcommittee at Japanese Society for Radiation Oncology. The Lung Cancer Working Group extracted eight reports and compared their data with those of the PBT registry from May 2016 to June 2018. All the analyzed 75 patients aged ≤80 years underwent proton therapy (PT) with concurrent chemotherapy for inoperable stage III NSCLC. The median follow-up period of the surviving patients was 39.5 (range, 1.6–55.6) months. The 2- and 3-year overall survival (OS) and progression-free survival rates were 73.6%/64.7% and 28.9%/25.1%, respectively. During the follow-up period, six patients (8.0%) had adverse events of Grade ≥ 3, excluding abnormal laboratory values. These included esophagitis in four patients, dermatitis in one and pneumonitis in one. Adverse events of Grade ≥ 4 were not observed. The results of these PBT registry data in patients with inoperable stage III NSCLC suggest that the OS rate was at least equivalent to that of radiation therapy using X-rays and that the incidence of severe radiation pneumonitis was low. PT may be an effective treatment to reduce toxicities of healthy tissues, including the lungs and heart, in patients with inoperable stage III NSCLC.

## INTRODUCTION

Photon-based radiation therapy with concurrent chemotherapy is recommended for inoperable stage III non-small cell lung cancer (NSCLC) [1–4]. However, for patients with unresectable stage III NSCLC, long-term survival remains at ~30% [5, 6]. Disease recurrence, particularly local and regional, remains problematic, and more effective treatments are actively being sought. Recently, consolidation treatment with human monoclonal antibodies against programmed cell death-ligand 1 (PD-L1) has been recommended in patients without disease progression and symptomatic radiation pneumonitis after chemoradiotherapy [7–9]. One approach for improved therapeutic efficacy currently being investigated is particle beam therapy (PBT), including proton beam therapy (PT) and carbon-ion therapy, which has been shown to affect target tissue while reducing the damage to surrounding healthy organs [10–16]. Particularly with PBT, the therapeutic ratio might be improved by escalating the radiation dose to control the tumor while decreasing toxicity, even in patients with concurrent chemotherapy.

Previous studies have reported that the 2-year survival rates of PBT with concurrent chemotherapy for unresectable stage III NSCLC are ~50–60% [17–24]. The frequency of pneumonitis of Grade ≥ 3 varies from 0 to 13% [17–24]. There have been few reports of PBT with concurrent chemotherapy for unresectable stage III NSCLC. Therefore, satisfactory results of PBT with concurrent chemotherapy for unresectable stage III NSCLC have not yet been obtained.

In May 2016, a nationwide multi-institutional cohort study on PBT started in all Japanese centers providing proton or carbon-ion radiation therapy. Since then, PBT can be applied in Japan as advanced medical care to patients with NSCLC. Recently, a Lung Cancer Working Group in the Particle Beam Therapy Committee and Subcommittee at Japanese Society for Radiation Oncology (JASTRO) comprehensively analyzed PT effects in patients with lung cancer. This working group comprises radiation oncologists of the JASTRO, oncologists of the Japan Society of Clinical Oncology and biostatisticians. Herein, we present the results of the investigations on patients with unresectable stage III NSCLC based on the Japanese multi-institutional registry dataset.

## MATERIALS AND METHODS

### Ethical approval

The protocol of this multi-institutional prospective study was approved by the Institutional Review Board of each participating institution before study initiation. Patients were informed of the concept, methodology and rationale of the treatment, which was performed in accordance with the 1983 amendment of the Declaration of Helsinki. Written informed consent was obtained from all patients.

### Eligibility criteria

**Fig. 1:**
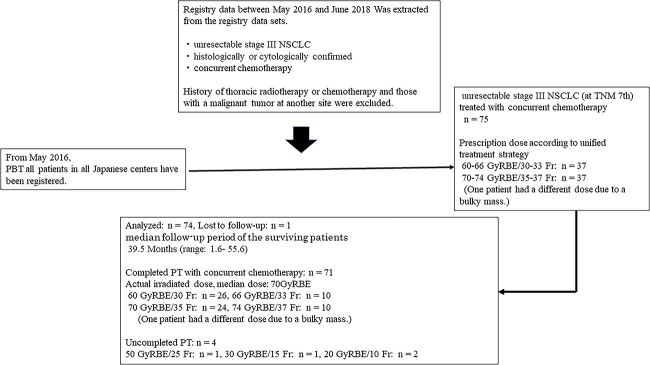
The flowchart of the registry data set.

The inclusion criteria were patients with unresectable, histologically or cytologically confirmed stage III NSCLC (according to the tumor, node, metastasis [TNM] classification of tumors, 7th edition [25]). PT data have been recorded for all patients in Japan. The clinical results of consecutive patients treated with concurrent chemotherapy between May 2016 and June 2018 were analyzed in this study (Fig. 1).

Patients who had a history of thoracic radiotherapy or chemotherapy and those with a malignant tumor at another site were excluded.

### Treatments

Dose fractionation and concurrent chemotherapy were performed according to a unified treatment strategy, which was based on JASTRO-approved guidelines [26]. In addition, chemotherapy was performed based on the standard of care for inoperable stage III NSCLC. All patients were presented at multidisciplinary team in the Cancer Board meetings of each institution. Based on the joint recommendations of the multidisciplinary team in the Cancer Board meetings, patients were indicated for PT with concurrent chemotherapy. Prescription doses were selected from 60 to 66 GyRBE (standard dose) or 70 to 74 GyRBE (high dose) in two GyRBE daily fractions for each patient at each institution (Fig. 1, Table 1). The extent of irradiation to the lymph node area was determined at the discretion of the radiation oncologist in charge at each institution, since the unified treatment policy does not stipulate elective nodal irradiation (ENI) / involved field irradiation for irradiation to the lymph node areas.

**Table 1:** Institutions and prescription doses

Institutions	60–66 GyRBE (*n*)	70–74 GyRBE (*n*)	NA (*n*)
A	0	1	1
B	0	10	0
C	2	0	0
D	0	19	0
E	17	0	0
F	4	5	0
G	0	2	0
H	14	0	0

Prescription doses were selected from 60 to 66 GyRBE (standard dose) or 70 to 74 GyRBE (high dose) in two GyRBE daily fractions for each patient at each institution.

### Evaluation and follow-up

Overall survival (OS) was defined as the time from the first day of PT to death from any cause. Progression-free survival (PFS) was defined as the time from the first day of PT to locoregional failure, distant tumor progression or death from any cause. Primary tumor recurrence was considered an event for evaluating local control. After treatment, OS, local control and PFS were evaluated every 3–6 months. Clinical examination and imaging studies (magnetic resonance imaging, computed tomography or positron emission tomography-computed tomography) were performed as appropriate in each institution. Patients with only Karnofsky performance status (PS) in this registry data were tabulated after being converted to Eastern Cooperative Oncology Group PS.

### Toxicity assessment

Acute toxicities were evaluated with the highest scores in the period from the start of PT up to 90 days according to the Common Terminology Criteria for Adverse Events, version 4.03. Late toxicities were graded 90 days after the commencement of PT according to the Common Terminology Criteria for Adverse Events, version 4.03 [27].

### Systematic review of PBT for inoperable stage III NSCLC

The systematic literature review on inoperable stage III NSCLC was conducted by the Lung Cancer Working Group in accordance with the Preferred Reporting Items for Systematic Review and Meta-Analyses guidelines (Fig. S1 and Table S1) [28]. PubMed was searched in 2017 and 2020 for clinical scientific reports on X-RT/X-IMRT/proton therapy/carbon ion radiation therapy and inoperable stage III NSCLC. In each team evaluating proton therapy and carbon ion radiation therapy for inoperable stage III NSCLC, two radiation oncologists independently reviewed the retrieved articles and selected potentially relevant articles based on titles and abstracts. If necessary, the two experts manually searched for other relevant articles. Afterward, full-text reviews identified studies that met the selection criteria established in each team assessing proton therapy and carbon ion radiation therapy.

Finally, five studies conducted before 2017 in patients with inoperable stage III NSCLC (5 single-center prospective studies) and three studies between 2017 and 2020 for proton therapy (2 single-center prospective studies, 1 retrospective study) were adopted [17–24].

### Statistical analysis

The survival probability after commencing treatment was estimated using the Kaplan–Meier method, and the level of significance was assessed using the log-rank test. The Kaplan–Meier curves were calculated using commercial software (R, version 4.2.2). A *P*-value <0.05 was considered statistically significant.

## RESULTS

The patients’ characteristics before treatment are summarized in Tables 2 and 3. From May 2016 to June 2018, 75 consecutive patients with inoperable stage III NSCLC (IIIA/IIIB: 46.7%/53.3%) were treated with PT and concurrent chemotherapy at multiple institutions in Japan. The median age was 66 (range, 34–80) years, and the cohort comprised 57 men and 18 women. All patients had a good PS (0 or 1). NSCLC was histologically confirmed in 74 patients, and only one patient was diagnosed cytologically.

**Table 2:** Patient characteristics

Characteristic	Value
Median age, years (range)	66 (34–80)
Sex, *n* (%)	
Male:female	57 (76):18 (24)
PS, *n* (%)	
0:1	61 (81.3):14 (18.6)
Histology, *n* (%)	
Adenocarcinoma	42 (56)
Squamous cell carcinoma	27 (36)
Unclassified NSCLC	5 (6.7)
Histology unproved	1 (1.3)
UICC seventh stage, *n* (%)	
IIIA:IIIB	35 (46.7):40 (53.3)
T-stage, *n*	
0:1:2:3:4	3:15:22:16:19
N-stage, *n*	
0:1:2:3	5:7:28:35
Primary site, *n*	
Upper:middle:lower	41:5:27
Others	2
Central:peripheral	23:51
Others	1
Total dose, *n* (%)	
60–66 GyRBE/30–33 Fr	37 (49.3)
70–74 GyRBE/33–37 Fr	37 (49.3)
Others	1 (1.3)
Irradiation technique, *n* (%)	
Broad beam:scanning	67 (89.3):8 (10.7)
Median follow-up time, months	39.5 (range: 1.6–55.6)
Interstitial pneumonia, *n*	5
Diabetes, *n*	12
Smoking history, *n*	
Nonsmoker	10
Smoker (Brinkman index < 600)	16
Smoker (Brinkman index ≥ 600)	49

UICC, Union for International Cancer Control [25].

**Table 3:** Patient characteristics by dose

Characteristic	Value
UICC seventh stage, *n*	
● 60–66 GyRBE/30–33 Fr	37
IIIA:IIIB	19:20
T-stage, *n*	
X:0:1:2:3:4	1:2:10:12:5:7
N-stage, *n*	
0:1:2:3	2:1:17:17
Primary site, *n*	
Upper:middle:lower:others	17:3:15:2
Central:peripheral:others	10:26:1
● 70–74 GyRBE/33–37 Fr	37
IIIA:IIIB	16:21
T-stage, *n*	
0:1:2:3:4	0:5:10:10:12
N-stage, *n*	
0:1:2:3	3:5:11:18
Primary site, *n*	
Upper:middle:lower:others	24:2:11:0
Central:peripheral:others	12:25:0

Fr, fraction, UICC, Union for International Cancer Control [25].

The selected irradiation dose in each institution was 60–66 and 70–74 gray radiobiological equivalent (GyRBE) in 37 and 37 patients, respectively. A different dose (other) was selected due to a bulky mass in one patient. Of the 75 patients, 71 completed PT. The actual irradiated dose was the median dose: 70 GyRBE, 34 patients in the high-dose group (74 GyRBE: 10 patients, 70 GyRBE: 24 patients) and 36 patients in the standard dose group (66 GyRBE: 10 patients, 60 GyRBE: 26 patients). Of four patients with uncompleted PT, the actual irradiated dose was 50, 30 and 20 GyRBE in one, one and two patients, respectively (Fig. 1).

Of the four patients who could not complete PT, two were unable to complete treatment due to deterioration of lung cancer, and the other two were unable to complete treatment due to adverse events. The remaining patients completed all the treatments. A total of 32 patients died, and one was unknown during the final follow-up. Of the 32 deceased patients, 27 died from the disease, and the other 5 died from other diseases or unknown causes. The median OS was 41.0 months.

**Fig. 2:**
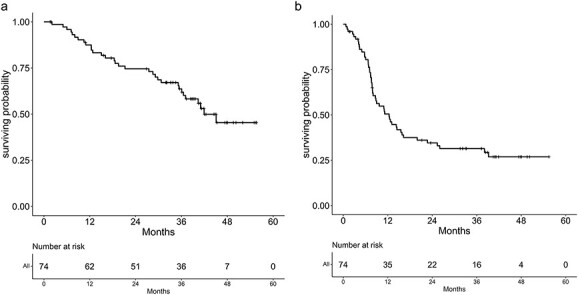
Kaplan–Meier curves of OS (**a**) and PFS (**b**) in all 74 patients.

**Fig. 3:**
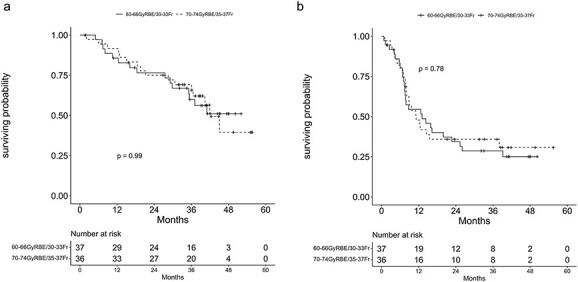
Kaplan–Meier curves of OS (**a**) and PFS (**b**) in each dose (60–66 GyRBE: 37 patients, 70–74 GyRBE: 36 patients).

Finally, 74 patients were analyzed, excluding the unknown case. The median follow-up period of the surviving patients was 39.5 (range, 1.6–55.6) months. The 2- and 3-year OS rates were 73.6 (95% confidence interval [CI], 63.0–86.0%) and 64.7% (95% CI, 53.3–78.4%), respectively. The median PFS was 10.9 months, and the 2- and 3-year PFS rates were 28.9 (95% CI, 19.1–43.6%) and 25.1% (95% CI, 15.9–39.6%), respectively (Fig. 2). In addition, the OS and PFS were also evaluated in terms of primary tumor stage, nodal stage, dose, occupied primary site and histology. Regarding the primary site, the PFS was significantly lower in the lower lobe compared with other primary sites (*P* = 0.0051). Similarly, the OS tended to be low at the lower lobe (*P* = 0.081). No significant difference was observed in the other analyses (Fig. 3 and Table 4).

**Table 4:** Univariate analyses for OS and PFS

Factors	*N* (%)	OS *P*-value	PFS *P*-value
Primary tumor			
T0–2	38 (52%)	0.14	0.66
T3–4	35 (48%)		
Regional lymph nodes			
N0–1	12 (16%)	0.68	0.28
N2	27 (36%)		
N3	35 (47%)		
Primary tumor site			
Lower lobe	26 (35%)	0.081	0.0051
Other sites	48 (65%)		
Pathology			
Adenocarcinoma	42 (57%)	0.95	0.69
Squamous cell carcinoma	26 (35%)		
Other pathologies	6 (8%)		

Six patients (8.0%) had adverse events of Grade 3. The Grade 3 esophagitis in three patients and Grade 3 pneumonitis in one patient were observed in the standard dose group, whereas in the high-dose group, Grade 3 esophagitis was observed in one patient. There was Grade 3 dermatitis in the different dose (other) patient due to a bulky mass. Adverse events of Grade ≥ 4 were not observed.

Local recurrence, regional lymph node recurrences and distant metastases are summarized in Table 5. Recurrence patterns were also examined by dose and by primary site. In all patients, there were 10 lymph node recurrences, six of which were outside the irradiation field. All isolated distant failures were observed in 29 patients, and three patients developed combined failures. Most patients who relapsed received additional therapy (48/54 patients) during the follow-up period.

**Table 5:** Failure pattern

	Value
Local recurrence, *n*	15
60–66 GyRBE/30–33 Fr, *n*	
T0:T1:T2:T3:T4	1:1:2:3:1
70–74 GyRBE/33–37 Fr, *n*	
T0:T1:T2:T3:T4	0:1:1:2:3
Regional lymph node recurrence, *n*	10
Within the irradiation field	6
Outside the irradiation field	4
Distant metastasis, *n*	29
Recurrence by primary site	
● Lower lobe, *n*	26
Local recurrence	4
Regional lymph node recurrence	
Within the irradiation field	5
Outside the irradiation field	2
Distant metastasis	12
● Other primary sites, *n*	48
Local recurrence	11
Regional lymph node recurrence	
Within the irradiation field	1
Outside the irradiation field	2
Distant metastasis	17

Fr, fraction; T, primary tumor stage [25].

## DISCUSSION

These are the first data of a Japanese nationwide multi-institutional prospective study of patients with inoperable stage III NSCLC undergoing PT. In this study, we found that PT with concurrent chemotherapy for inoperable stage III NSCLC had manageable toxicity and encouraging OS rates. In addition, there was no difference in local control and complications in both the standard dose and high-dose groups. In this study, the 3-year OS and PFS rates in the 74 patients were 64.7 (95% CI, 53.3–78.4%) and 25.1% (95% CI, 15.9–39.6%). The median OS and PFS were 41.0 and 10.9 months, respectively, and the toxicities were considered acceptable.

Treatment outcomes of systematic literature review on unresectable stage III NSCLC described the combined use with chemotherapy. In photon RT, the RTOG 0617 study, which was the best clinical data before the introduction of PD-L1 inhibitors, the median OS and PFS were 28.7 and 12.0 months, respectively [10–12]. In the PACIFIC study, in which patients were treated with consolidation durvalumab, following definitive therapy for inoperable stage III NSCLC, the subsequent 3-year OS rate was reported to be 57%, and the median PFS was 16.8 months [7–9]. In the literature, three PT studies were single-center prospective trials [17, 18, 20]. Oshiro *et al*. [18] reported that PT with concurrent chemotherapy for unresectable stage III NSCLC achieved favorable survival (2-year OS rate, 51%; mean, 26.7 months) with tolerable toxicities. Nguyen *et al*. [17] reported a 3-year OS rate of 41.0% for stage IIIA NSCLC and 44.5% for stage IIIB. Chang *et al*. [20] reported 5-year OS and PFS rates of 29 and 22%, respectively. Treatment outcomes of this study were at least equivalent to that of radiation therapy using X-rays and PT with concurrent chemotherapy.

Patients receiving definitive chemoradiotherapy for inoperable stage III NSCLC are at significant risk of developing treatment-related thoracic toxicities, including pneumonitis, carditis and esophagitis. Particularly, radiation pneumonitis is often described as an adverse event. In this study, six patients (8.0%) had adverse events of Grade ≥ 3, including Grade 3 pneumonitis in one patient. In systematic literature review on treatment outcomes of unresectable stage III NSCLC, which described the combined use of PBT with chemotherapy, the frequency of pneumonitis of Grade ≥ 3 varies from 0 to 13% [17–24]. It was thought that treatment-related toxicities observed in this study were mild. Similarly, the PACIFIC study reported radiation pneumonitis of Grade ≥ 3 of 4.4% in the consolidation durvalumab group. Furthermore, in the PACIFIC study, intensity-modulated radiation therapy (IMRT) was used as a modern technique that provides excellent target volume coverage and reduces high doses to organs at risk, but this technique increases the size of low-dose areas, including the lungs [10–12]. A large low-dose irradiated area, such as V5 in the lungs, has been reported to be associated with symptomatic radiation pneumonitis [29]. PT has demonstrated encouraging clinical outcomes for inoperable stage III NSCLC with less toxicity than IMRT [17, 20, 30]. It may be particularly beneficial in reducing low-dose areas, such as V5 and symptomatic radiation pneumonitis [31]. On the other hand, a randomized controlled study comparing intensity-modulated proton therapy and IMRT revealed no significant benefits regarding the occurrence of radiation pneumonitis [22]. Further examination is necessary in the future.

After chemoradiotherapy, 30–40% of patients develop locoregional tumor recurrence [10–12]. Therefore, irradiated dose escalation for inoperable stage III NSCLC has been attempted, but the results were unsatisfactory [10–12]. In this study, 15 patients (20.0%) had local recurrence; 8 and 7 in the standard dose and high-dose groups, respectively (Table 5). Furthermore, prescription doses were selected from 60 to 66 GyRBE (standard dose) or 70 to 74 GyRBE (high dose) in two GyRBE daily fractions for each patient at each institution (Table 1). Similarly, ENI/IF of lymph node irradiation was also determined for each patient at each institution. There was no difference in local control and OS rates in both groups. However, no increase in complications occurred even in the high-dose group; therefore, it is considered that the high dose using PT was safe and feasible. Considering the outcomes of systematic literature review, patients in the three studies in whom 74 GyRBE was prescribed showed no improvement in OS [17-18, 20]. Therefore, dose escalation alone may not improve treatment outcomes in unresectable stage III NSCLC, suggesting the need for multidisciplinary approaches, such as immunotherapy, including consolidation durvalumab, following definitive therapy. The need for local dose escalation seems to require further investigation in conjunction with consolidative immunotherapy. We consider it necessary to examine in future studies the optimal dose in each tissue, appropriate lymph node irradiation area and the significance of increasing the local dose and efficacy of consolidative immunotherapy, including durvalumab, following definitive therapy.

The RTOG 0617 study identified the cardiac radiation dose (heart V40) as an independent predictor of OS [10–12]. Moreover, Chang *et al*. [20] reported tumor location effects on OS. Their study results suggested that left-sided and right lower lobe disease was associated with worse OS, which might be related to the proximity to the heart [20]. The OS of the lower lobe primary site tended to be worse than that of other lobes in this study (Table 4). However, the present analysis did not confirm an apparent effect of the tumor location on OS. On the other hand, PFS was significantly worse in the lower lobe (Table 4). In the lower lobe primary site, there were more regional lymph node recurrences outside the irradiation field than in other primary sites (Table 5). In fact, most patients who relapsed received additional therapy (48/54 patients), which was considered a contributing factor to the lack of significance in the OS rate. Besides, no increase in severe complications was observed in the lower lobe primary site in this study. Therefore, this was thought to be due to recurrence and metastasis rather than complications. Follow-up studies should examine the association between late complications and long-term outcomes.

This study has several limitations, including its limited sample size, resulting in inevitable selection bias. In addition, dose volume histogram (DVH) parameters of the lungs, heart and esophagus were not collected as registry data in this study; therefore, it could not be evaluated. Moreover, Grade 2 complications, including symptomatic pneumonitis, could not be evaluated, though symptomatic radiation pneumonitis (Grade ≥ 2) after chemoradiotherapy was an important exclusion criterion for the administration of maintenance PD-L1 inhibitors following definitive chemoradiotherapy in the PACIFIC study [7–9]. However, this was a prospective observational study, and the treatment protocol remained unchanged. We believe that our data are sufficiently reliable and may form the basis for future prospective clinical trials regarding targeted agents and immunotherapeutic approaches. Regarding DVH and symptomatic radiation pneumonitis, future studies with larger numbers of patients and longer follow-up periods are warranted to corroborate our findings.

## CONCLUSION

In conclusion PT with concurrent chemotherapy was feasible and safe in patients with inoperable stage III NSCLC undergoing definitive therapy. The results of these PBT registry data suggest that the OS rate was at least equivalent to that of radiation therapy using X-rays and that the incidence of severe radiation pneumonitis was low. PBT may be an effective treatment to reduce toxicities of healthy tissues, including the lungs and heart. Further improvement in treatment results is expected by consolidation durvalumab following definitive therapy. Therefore, it is also necessary to evaluate treatment outcomes with consolidation durvalumab following chemo-particle definitive therapy.

## DATA AVAILABILITY

Participants have not consented to have their data made public, so the data is not available.

## CONFLICT OF INTEREST

M. Satouchi received grants and a speaker honorarium from CHUGAI PHARMACEUTICAL CO., LTD, AstraZeneca K.K., ELI LILLY JAPAN K.K., ONO PHARMACEUTICAL CO., LTD., Bristol Myers Squibb, Merck & Co., Inc., Merck, Janssen Pharmaceutical K.K., Amgen Inc., TAIHO PHARMACEUTICAL CO., LTD., Pfizer Inc., DAIICHI SANKYO CO., LTD., Eisai Co., Ltd., Takeda Pharmaceutical Company Limited, and Novartis Pharma K.K. M Satouchi received grants from GlaxoSmithKline Consumer Healthcare Japan K.K. and Bayer Pharma Japan. H. Harada received a speaker honorarium from Hitachi, Ltd., AstraZeneca K.K., Brain Lab,Inc., Accuray Japan K.K., CHUGAI PHARMACEUTICAL CO., LTD., Eisai Co., Ltd., TAIHO PHARMACEUTICAL CO., LTD., Takeda Pharmaceutical Company Limited, Pfizer Inc., Merck & Co., Inc. and Nihon Medi-Physics Co., Ltd. K. Mori received a speaker honorarium from CHUGAI PHAEMACEURICAL CO., LTD., ONO PHARMACEUTICAL CO., LTD., DAIICHI SANKYO CO., LTD. and ELI LILLY JAPAN K.K.

## FUNDING

This work was supported by Hokkaido University (Functional enhancement promotion expenses by the Ministry of Education, Culture, Sports, Science and Technology) and AMED under Grant Number JP16lm0103004.

## PRESENTATION AT A CONFERENCE

None declared.

## Supplementary Material

Supplementary_data_1_detailed_description_about_SR_rrad017
