# Supplementary material for: Clinical results of proton beam radiotherapy for inoperable stage III non-small cell lung cancer: a Japanese national registry study
Source: J Radiat Res. 2023 May 3;64(Suppl 1):i8–i15. doi: 10.1093/jrr/rrad017 (PMC10278876; doi:10.1093/jrr/rrad017)
Supplement: Supplementary_data_1_detailed_description_about_SR_rrad017 [file supplementary_data_1_detailed_description_about_sr_rrad017.docx]

**Supplemental data 1. Detailed description of the method for systematic review**

This systematic review (SR) on particle beam therapy (PBT) for inoperable stage III non-small cell lung cancer (NSCLC) was conducted twice, in 2017 and 2020. In SR on PBT for inoperable stage III NSCLC at 2020, medical literature published in English was searched in PubMed using the terms in Table S1a. The inclusion criteria for the literature search were defined using the Population, Intervention, Control, Outcome, Study Design (PICOS) approach and shown in Table S1b. We defined the inclusion criteria as the following: clinical trials, prospective, and retrospective studies were eligible. Two radiation oncologists independently reviewed the retrieved articles and selected potentially relevant articles based on the titles and abstracts. After that, finally, full-text reviews were performed to identify studies fully meeting the selection criteria (Table S1b). PRISMA Flow Diagram of the SR on inoperable stage III NSCLC was illustrated in Figure S1. The same two and colleague extracted data from the identified studies which met fully selection criteria. The extracted variables included study-level or patient-level characteristics, such as study design, phase of trial, sample size, duration of follow-up, dose per fraction, fraction number. The information about the endpoint outcomes of progression free survival and overall survival and treatment-induced toxicities of grade ≥ 3 were also extracted. Finally, 5 studies conducted prior to 2017 in patients with inoperable stage III NSCLC (5 single-center prospective studies) and 3 studies between 2017 and 2020 (2 single-center prospective studies, 1 retrospective study) were adopted [18–25].

| Table S1a. Research terms for systematic review for inoperable stage III non-small cell lung cancer on particle beam therapy |
| --- |
| (("Lung Neoplasms"[mh] OR "Lung Neoplasms"[tiab] OR "Lung Neoplasm"[tiab] OR "Lung Cancer"[tiab] OR "Lung Cancers"[tiab] OR "Pulmonary Cancer"[tiab] OR "Pulmonary Cancers"[tiab] OR "Pulmonary Neoplasms"[tiab] OR "Pulmonary Neoplasm"[tiab])) AND (((("particle beam"[tiab] OR "particle radiations"[tiab] OR "particle radiation therapy"[tiab] OR "particle therapy"[tiab] OR "particle therapies"[tiab] OR "particle treatment"[tiab] OR "particle treatments"[tiab])) OR ("Protons/therapeutic use"[mh] OR "Proton Therapy"[mh] OR ((proton[tiab] OR Protons[tiab]) AND (treatment[tiab] OR treatments[tiab] OR therapy[tiab] OR therapies[tiab] OR therapeutic[tiab])))) OR ("Heavy Ion Radiotherapy"[mh] OR "Heavy Ions/therapeutic use"[mh] OR (("heavy Ion"[tiab] OR "heavy Ions"[tiab] OR "Carbon Ion"[tiab] OR "Carbon Ions"[tiab]) AND (treatment[tiab] OR treatments[tiab] OR Therapy[tiab] OR Therapies[tiab] OR therapeutic[tiab] OR Radiotherapy[tiab] OR Radiotherapies[tiab])))) |

| **Table S1b. Population, Intervention, Control, Outcome, Study Design (PICOS) inclusion criteria.** | |
| --- | --- |
| **Population** | Patients diagnosed with lung cancers. |
| **Intervention** | particle beam therapy |
| **Control** | Control group was not stipulated. |
| **Outcomes** | The study must report the following outcomes: progression free survival rate, overall survival rate, median survival time, incidence of any toxicity of grade ≥ 2. |
| **Study design** | For lung cancer treatment with particle beam therapy, clinical trials, prospective, and retrospective studies were eligible, although a study must include at least 30 patients which have received for stage III non-small cell lung cancer at first screening. Case reports were excluded.  For full-text articles assessed, clinical trials, prospective studies were eligible. Also, retrospective studies with multiple centers and many patients (>100 patients) were considered eligible. |

**Figure S1. Inclusion flow diagram for the selection of articles included in the systematic review of the particle beam therapy for lung cancers.**

**
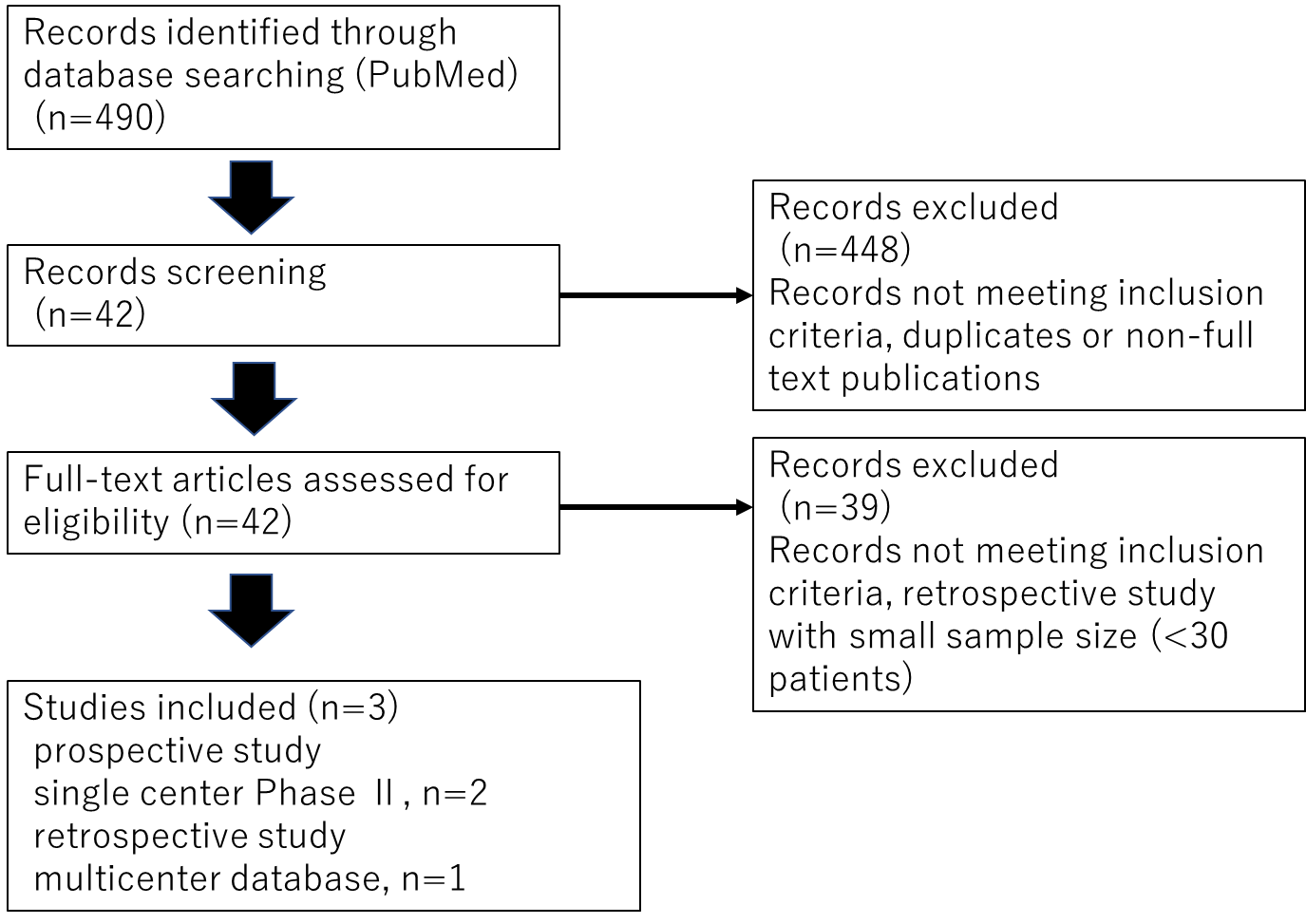
**
